# Expression of dynein, cytoplasmic 2, heavy chain 1 (DHC2) associated with glioblastoma cell resistance to temozolomide

**DOI:** 10.1038/srep28948

**Published:** 2016-07-04

**Authors:** Hai Wang, Wenfeng Feng, Yuntao Lu, Hezhen Li, Wei Xiang, Ziyang Chen, Minyi He, Liang Zhao, Xuegang Sun, Bingxi Lei, Songtao Qi, Yawei Liu

**Affiliations:** 1 Department of Neurosurgery, Nanfang Glioma Center, Nanfang Hospital, Southern Medical University, Guangzhou 510515, China; 2 Department of Organ Transplantation, Zhujiang Hospital, Southern Medical University, Guangzhou 510515, China; 3 Department of Pathology, Nanfang Hospital, Southern Medical University, Guangzhou 510515, China; 4 School of Traditional Chinese Medicine, Southern Medical University, Guangzhou 510515,China; 5 Nanfang Neurosurgery Research Institution, Nanfang hospital, Southern Medical University, Guangzhou 510515,China

## Abstract

Temozolomide (TMZ) is the main chemotherapeutic drug utilized for the treatment of glioblastoma multiforme (GMB), however, drug resistance often leads to tumor recurrence and poor outcomes. GMB cell lines were treated with TMZ for up to two weeks and then subjected to proteomics analysis to identify the underlying molecular pathology that is associated with TMZ resistance. Proteomics data showed that TMZ altered expression of proteins that related to cytoskeleton structure and function, such as DHC2 and KIF2B. qRT-PCR and immunofluorescence were used to verify expression of DHC2 and KIF2B in these cells. Immunohistochemistry was used to verify expression of these two proteins in xenografts of a nude mouse model, and *ex vivo* GBM tissue samples. Their expression was knocked down using siRNA to confirm their role in the regulation of GBM cell sensitivity to TMZ. Knockdown of DHC2 expression enhanced sensitivity of U87 cells to TMZ treatment. *Ex vivo* data showed that DHC2 expression in GBM tissue samples was associated with tumor recurrence after TMZ chemotherapy. These results indicated cytoskeleton related protein DHC2 reduced sensitivity of GBM cells to TMZ treatment. Further studies should assess DHC2 as a novel target in GBM for TMZ combination treatment.

Glioblastoma multiforme (GBM) is the most frequently diagnosed primary malignant brain tumor in adults^1^,^2^. Clinically, GBM is the most common and aggressive brain malignancy and incurable despite advancements in therapies, including neurosurgery, alkylating agent based-chemotherapy and radiation. Indeed, the median survival of GBM patients is approximately 15 months and the five-year survival is less than 10%^3^. Temozolomide (TMZ) is the most frequently used chemotherapeutic agent to treat GBM and a previous clinical trial of more than 500 participants showed that patients randomized to radiation plus TMZ chemotherapy had a median survival of 14.6 months versus 12.1 months in patients with radiotherapy alone^4^. This treatment regime has now become standarized therapy for GBM. The therapeutic benefit of TMZ depends on its ability to alkylate/methylate DNA, which most often occurs at the N^7^ or O^6^ positions of guanine residues. Methylation damages genomic DNA and triggers death of tumor cells. However, glioblastoma patients have a propensity to develop drug resistance during TMZ treatment as tumor cells gain the ability to repair DNA damage caused by TMZ, therefore diminishing the therapeutic efficacy of TMZ. This occurs due to expression of O^6^-alkylguanine DNA alkyltransferase (AGT) encoded in humans by the O^6^-methylguanine-DNA methyltransferase (MGMT) gene^5^. Although expression of the DNA repair protein MGMT has been generally accepted to play an important role in GBM resistance to TMZ, TMZ-resistant GBM tissue specimens have been shown to exhibit reduced MGMT expression in more than 50% of GBM cases; thus, the mechanism of TMZ resistance in GBM patients remains unknown. Assessment and identification of the underlying molecular events of TMZ resistance may, therefore, provide novel targets for treatment as well as elucidating the molecular factors involved in the progression of GBM.

Both cell mobility and the cytoskeleton have been reported to be associated with cancer progression and drug resistance. Our current study focused on DHC2 and KIF2B after proteomic analysis of TMZ-treated glioma cells. DHC2 (dynein, cytoplasmic 2, heavy chain 1, also known as DYNC2H1, DHC1b, DYH1B, DNCH2, or SRTD3) belongs to a member of cytoplasmic dynein protein family and is ubiquitously expressed in cells^6^. Dynein is a molecular motor in cells that converts chemical energy into mechanical force for cell mobility^7^. Dynein can also transport various cellular cargo by “walking” along cytoskeletal microtubules towards the minus-end of microtubules, leading to the cell center^8^ and this movement is known as retrograde intra-flagellar transport (IFT)^9^,^10^. Similarly, KIF2B (Kinesin family member 2B) is a member of kinesin family proteins and plays a role in cytoskeleton organization and cell division. In cells, kinesin moves along microtubule filaments through hydrolysis of ATP^11^,^12^,^13^. The movement of kinesin is necessary for a variety of cellular activities, such as mitosis, meiosis, and transportation of cellular cargo^14^. The temporal regulation of kinetochore-microtubule attachments by KIF2B, CLASP1, and Astrin plays a central role in correct chromosome segregation during cell division^15^.

Thus, in our current study, we performed a proteomic analysis using *in vitro* cultured GBM cells treated with 200 μM TMZ for up to two weeks and then confirmed expression of genes using qRT-PCR and immunofluorescence in cells, xenografts and *ex vivo* tissue samples. Following this, we then further focused on DHC2 and KIF2B and examining their role in mediation of TMZ resistance in GBM cells.

## Results

### TMZ reduced GBM cell viability, changed cell morphology and induced DNA damage response

Viability of U87 and U251 cells was reduced after treated with 200 μM TMZ at both one and two weeks compared to the DMSO-treated cells (Fig. S1a). Cell cycle analysis showed that U87 and U251 cells treated with TMZ arrested at G2 phase of the cell cycle (Fig. S2). Although most tumor cells had undergone apoptosis or were in the process of after two-week treatment with TMZ, a few surviving cells underwent cell cycle arrest at the G2 phase of cell cycle.

Morphology of cells was significantly altered after treatment with TMZ (200 μM, similarly hereinafter). As shown in Fig. S1b, after treatment of GBM cells with TMZ for a week, the morphology of surviving cells changed significantly: the bodies and nuclei of cells became much larger and almost all surviving cells had extending long protrusions from their cell bodies. Most cells had two protrusions extended from the two poles of the cell body, and some cells had three or more protrusions. The protrusions were of different lengths, widths and shapes and some extended from the same cell body (Fig. S1b). TMZ-treated cells were different in morphology from DMSO controls; there were vesicles in cell bodies that are usually around the nuclei in the majority of these cells after TMZ treatment (Fig. S1b).

For two weeks treated with TMZ, cell size and nuclei were much larger compared to one-week of TMZ treatment and cell body protrusions were significantly longer (Fig. S1b). Cells had more protrusions with some having four or more protrusions. Some giant tumor cells had more than ten protrusions. Protrusions were able to connect with each other more easily than cells after one-week treatment. These changes in cell modality indicated re-organization of the cytoskeleton in cells.

**Figure 1:**
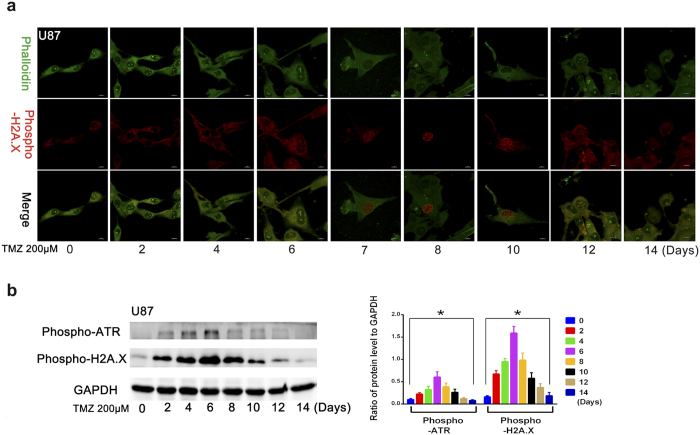
Effects of temozolomide on DNA damage response in GBM cells. (**a**) Immunofluorescence analysis of the level of phospho-H2A.X in nucleus which can reflect DNA damage response increased significantly at day 6, peaked during days 7–10 and decreased significantly at day 12 after treatment temozolomide (200 μM). (**b**) The level of phospho-H2A.X and phospho-ATR in cells, both of which reflect DNA damage responses, increased immediately after TMZ treatment, and the expression level of both phospho-H2A.X and phospho-ATR peaked at day 6 after TMZ treatment. Thereafter, DNA damage response reduced (*P < 0.05).

In addition, we assessed DNA damage and found that the level of phospho-H2A.X in cell nuclei reflected DNA damage response and increased significantly at approximately 6 days and peaked at 7–10 days, and returned to baseline 12 days after treatment with TMZ (Fig. 1a). Furthermore, the level of phospho-H2A.X and phospho-ATR in cell also reflected DNA damage response and were increased immediately following TMZ treatment, with their expression level peaking at 6 days after TMZ treatment (Fig. 1b). This finding indicates that the DNA damage response could be induced at 2 days after 200 μM TMZ treatment and increased continuously, reaching a peak 6–8 days after treatment, and then returned to baseline.

### Identification of differentially expressed proteins after TMZ treatment

**Figure 2:**
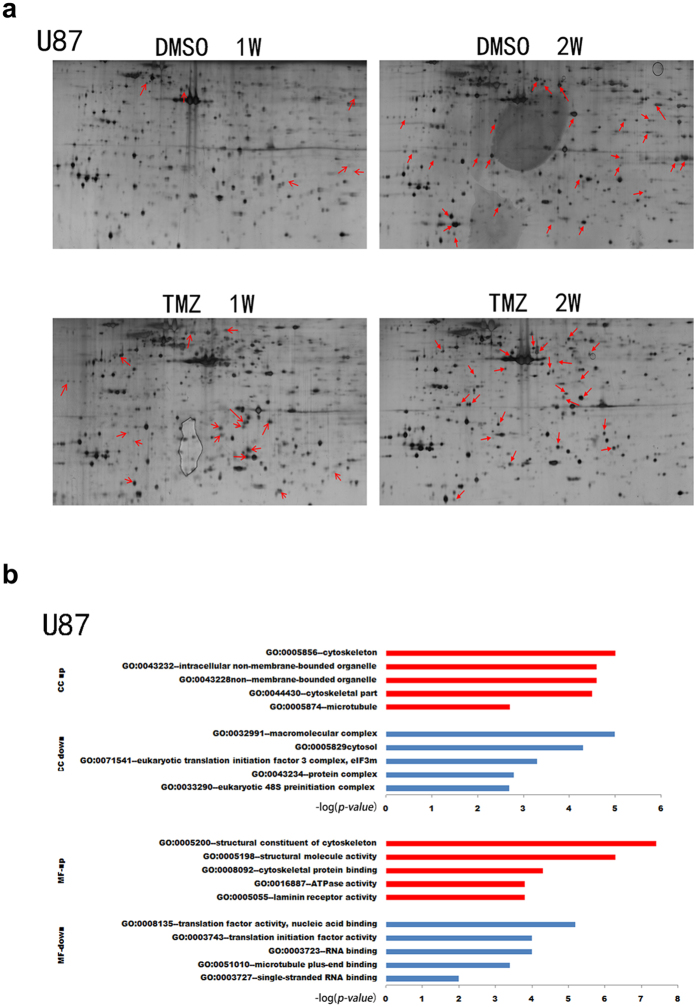
Identification of differentially expressed proteins after TMZ treatment of GBM cells. (**a**) The 2-DE data of U87 cells after treated with temozolomide for a week or two. Differentially expressed protein spots were noted with red arrows. (**b**) Gene ontology analysis with Enrichment analysis (beta) (http://geneontology.org/). Upper, the top 5 dysregulated cellular component (CC) ranked according to –log (p-value), *P* < 0.05. Lower, the top 5 dysregulated molecular functions (MF) ranked according to –log (p-value), *P* < 0.05.

Next, we performed proteomic analysis to identify differentially expressed proteins after TMZ treatment using 2-D gel (Fig. 2a) and MALDI-TOF/TOF MS analyses (Fig. 2b). PDQuest software analysis revealed 47 proteins that were up-regulated or down-regulated by two-fold after TMZ treatment (Tables S2 and S3). Specifically, a broadly adopted web-based functional analysis and GO analysis were used to derive biological meaning from proteomics data. The top 5 regulated molecular functions (MF) and cellular component (CC) of up-regulated proteins in rank were mainly related to cytoskeleton organization, such as structural constituents of the cytoskeleton, cytoskeletal protein binding, laminin receptor activity, cytoskeleton itself, cytoskeletal components, and microtubules (Fig. 2b).

### Cytochalasin D (CCD) increased sensitivity of U87 cells to TMZ

Since TMZ resistance was correlated with altered expression of cell cytoskeleton-related proteins, we assessed whether agents that induced cytoskeleton damage, such as cytochalasin D (CCD), could induce TMZ sensitivity in cells. After treatment with Cytochalasin B (CCB) 1 μg/mL), CCD (0.5 μg/mL), or nocodazole (1 μg/mL) in combination with TMZ (200 μM) for either one week or two, cell morphology changed significantly (Fig. S3). Cell morphology was altered after CCB or CCD treatment, with most cells increasing in size and displaying more protrusions compared to TMZ-only treated cells. However, there was no significant difference in morphology between cells treated with CCB+TMZ or CCD+TMZ and the TMZ group. However, treatment with nocodazole, whether alone or in combination with TMZ, aused cells to become significantly rounded in shape without any protrusions, with a significant number of vesicles in cytoplasma (Fig. S3a). Viability of TMZ+ CCD treated cells was significantly reduced compared to that of TMZ-only treated cells or CCD+ DMSO cells. CCD increased the sensitivity of U87 cells to TMZ (Fig. S3b), however sensitivity remained unchanged in CCB+TMZ and nocodazole+ TMZ treated cells.

### TMZ up-regulated expression of DHC2 and KIF2B in U87 cells and tumor xenografts

**Figure 3:**
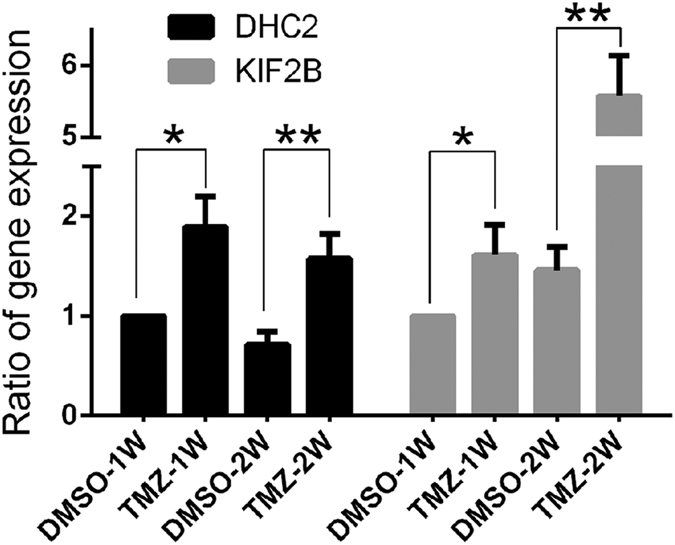
qRT-PCR validation of DHC2 and KIF2B expression in temozolomide-treated cells. Expression of DHC2 and KIF2B mRNA was detected in GBM cells treated with 200 μM temozolomide or DMSO for one to two weeks using qRT-PCR (*P < 0.05 and **P < 0.01).

**Figure 4:**
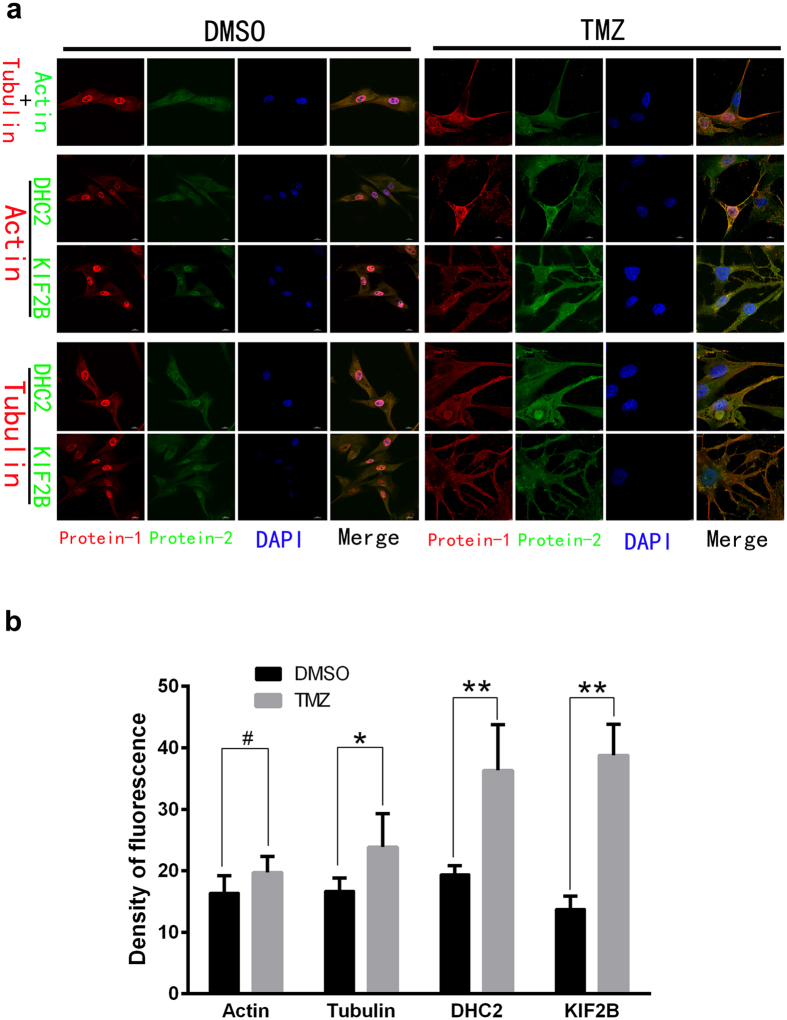
Effects of temozolomide on regulation of skeleton and skeleton-related protein expression in GBM cells. (**a**) Rearrangement and damage of skeleton and immunofluorescence analysis of Actin, Tubulin, DHC2 and KIF2B in U87 cells after treatment with temozolomide/DMSO for two weeks. Magnification, ×1200. (**b**) Summarized data of immunofluorescence about these proteins (^#^*P* > 0.05; *P < 0.05; and **P < 0.01).

Our data revealed that two cytoskeleton-related proteins, DHC2 and KIF2B, were implicated in the effects of TMZ treated cells. We then verified their expression using qPCR and immunofluorescence. As showed in Fig. 3, DHC2 and KIF2B proteins in TMZ-treated GBM cells for a week or two were significantly up-regulated compared to vehicle control. Immunofluorescence data further approved these findings (Fig. 4b). Moreover, DHC2 and KIF2B proteins were overexpressed in the TMZ group and associated with cytoskeleton rearrangement (Fig. 4a). Indeed, cell size and nuclei in TMZ-treated cells were much larger compared to controls with significant differences in morphology. TMZ treatment induced the formation of vacuoles in the cytoplasm without cytoskeleton arrangement. These results suggest that actin microfilaments were significantly re-organized, with an absence of continuous microfilaments. Microtubules that mainly consist of tubulin were rearranged and redistributed from the cytoplasm to the edge of cells, especially near to protrusions. DHC2 and KIF2B proteins were co-localized with tubulin and actin, respectively. In addition, fluorescence intensity of DHC2 and KIF2B expression in TMZ-treated cells was much higher than that in control cells (Fig. 4). These data are consistent with the data shown in Table S2.

**Figure 5:**
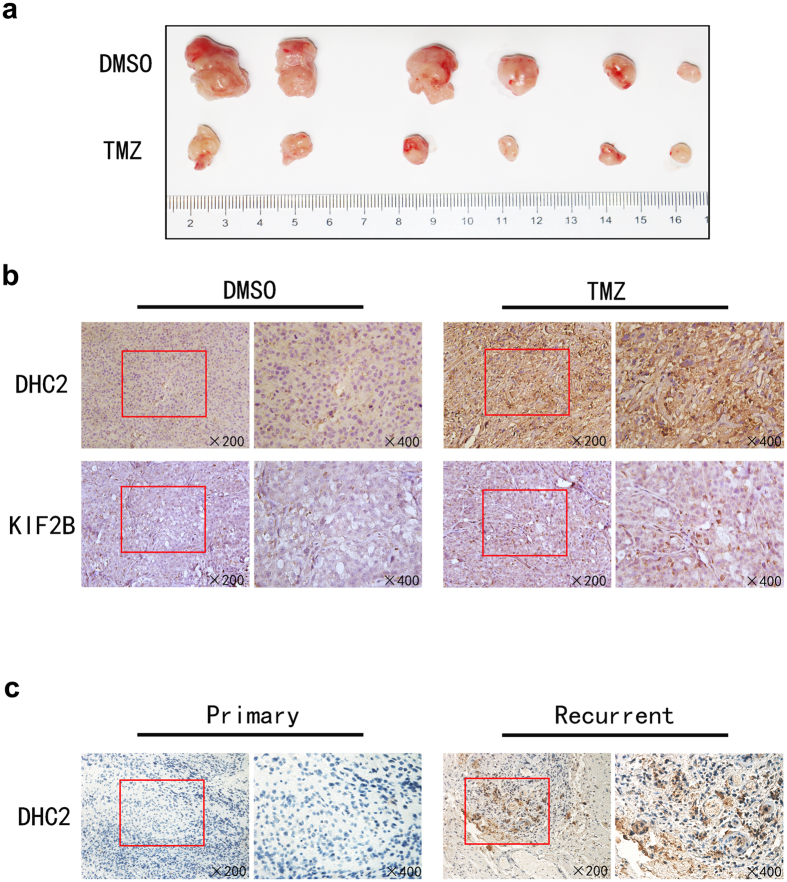
Expression of DHC2 and KIF2B in nude mouse tumor xenografts. (**a**) Tumors samples from xenograft model. (**b**) Immunohistochemistry analysis of DHC2 and KIF2B in tissue samples from xenograft models. (**c**) Expression of DHC2 in GBM tissue specimens. Immunohistochemical analysis of DHC2 in primary and recurrence GBM tissue samples.

Tumor xenografts also demonstrated DHC2 and KIF2B up-regulation following systemic treatment of mice with TMZ. After treatment with TMZ for 2 weeks, DHC2 and KIF2B expression was up-regulated in tumor xenografts (Fig. 5b).

### High expression of DHC2 in recurrent GBM tissue samples after TMZ treatment

To confirm our current *in vitro* and xenograft data, we immunostained DHC2 and KIF2B proteins in 9 cases of GBM tissue samples from patients with recurrent GBM who were treated with TMZ before their second surgery, and in 12 samples of primary GBMs without TMZ treatment. As shown in Fig. 5c, expression of DHC2 in the recurrent cases was higher than the GBM tissues without TMZ treatment, whereas there was no significant difference in KIF2B expression between these two groups of tissue samples.

### Knockdown of DHC2 expression enhanced sensitivity of U87 and primary GBM cells to TMZ

**Figure 6:**
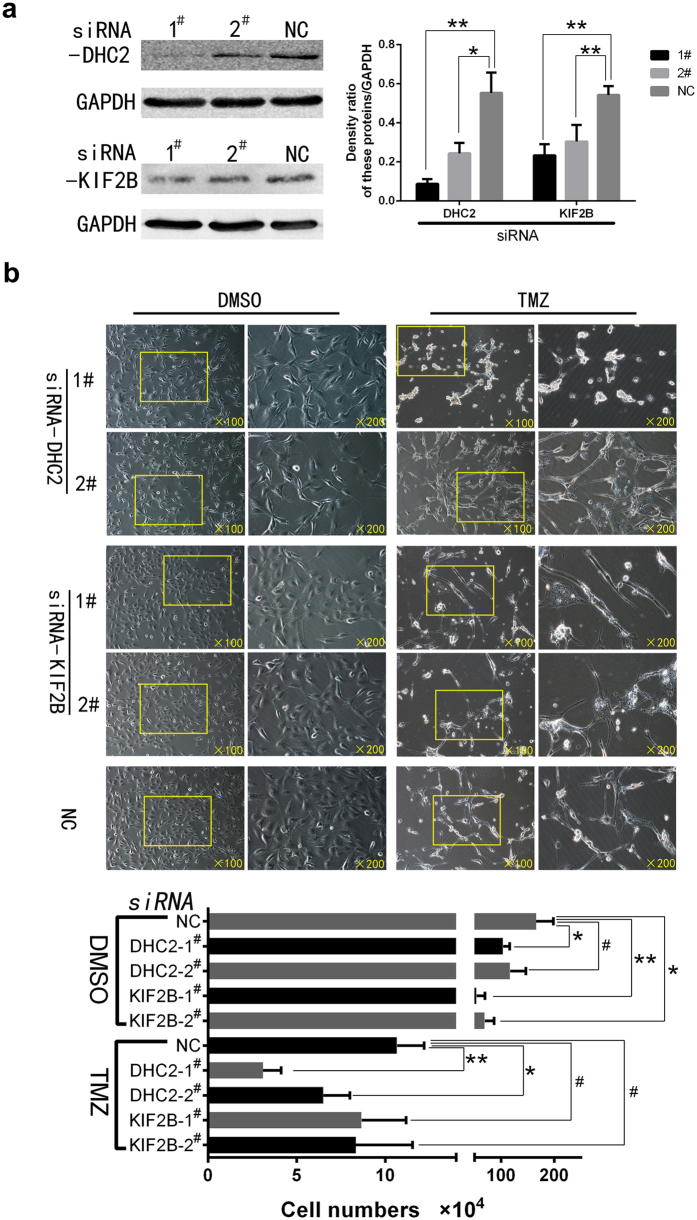
Knockdown of DHC2 and KIF2B expression in U87 cells. (**a**) Western blot. Cells were grown and transfected with two different siRNAs for 8 h and cultured for 48 h and then subjected to Western blot analysis for protein expression. The graph is summarized data of the blot. *P < 0.05 and **P < 0.01. (**b**) Morphology and viability change of cells after knockdown of DHC2 and KIF2B expression followed by temozolomide treatment for one week. Cells were then counted and the graph shows the number of surviving cells in different groups (^#^*P* > 0.05; *P < 0.05 and **P < 0.01).

We then sought to knock-down DHC2 and KIF2B expression in U87 cells using siRNA and then treated these cells with TMZ and subjected cells to cell viability and morphology analysis (Fig. 6). The data showed that viability of DHC2-knocked down cells treated with TMZ reduced significantly, in particular with #1 siRNA, whereas there was no significant effect in KIF2B-knocked down cells treated with TMZ (Fig. 6c). The morphology of DHC2-knocked down cells after treatment with TMZ was different from control cells without DHC2 knockdown also treated with TMZ, in particular, using #1 siRNA. DHC2-knocked down cells were small and smooth compared to negative control cells after TMZ treatment (Fig. 6b).

**Figure 7:**
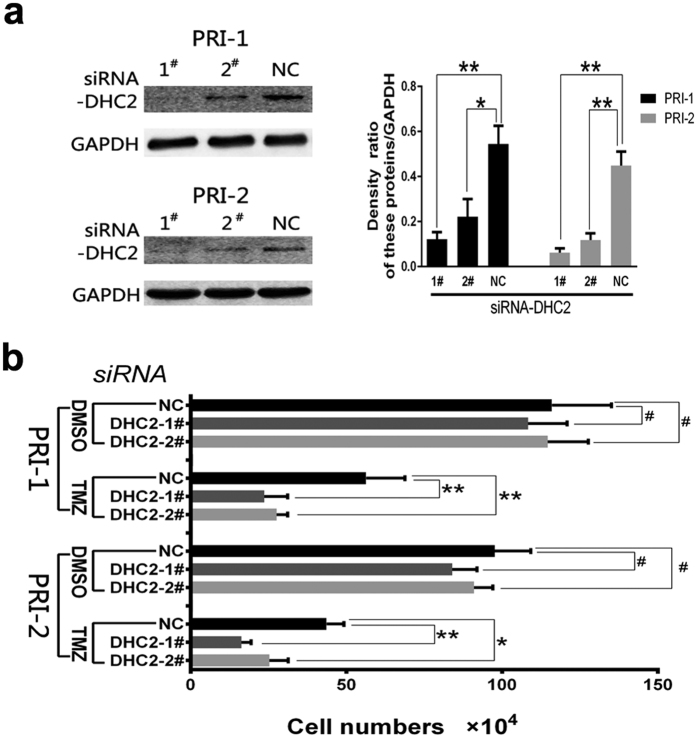
Knockdown of DHC2 expression in 2 different primary GBM cell lines. (**a**) Western blot. Cells were grown and transfected with two different siRNAs for 8 h and cultured for 48 h and then subjected to Western blot analysis of protein expressions. The graph is the summarized data of the blot. *P < 0.05; **P < 0.01. (**b**) Changes in viability of cells after knockdown of DHC2 expression followed by temozolomide treatment for one week. Cells were then counted and the graph showed the number of survival cells in different groups (^#^*P* > 0.05; *P < 0.05; and **P < 0.01).

To verify the results in U87 and U251 cell lines, 2 primary GBM cell lines were established from fresh clinical GBM patient tumor samples. Knockdown of DHC2 expression was conducted in primary GBM cell lines with two different siRNAs and then cells were subjected to Western blot analysis in order to examine protein expression (Fig. 7a). Following knock-down, numbers of surviving cells in the different experimental groups were counted (Fig. 7b). The data showed that knockdown of DHC2 expression enhanced sensitivity of primary GBM cells to TMZ (Fig. 7). These findings were consistent with those in U87 and U251 cell lines.

## Discussion

Clinically, GBM is challenging to treat because tumor cells are often resistant to conventional chemotherapy. Furthermore, the blood-brain barrier limits drug bioavailability. TMZ-containing chemoradiation therapy has become the standard treatment for GBM; however, resistance to TMZ results in treatment failure. Although previous studies sought to reveal the mechanisms underpinning TMZ resistance, much work has focused on comparing differences between GBM cells sensitivity and resistance to TMZ.

In our current study, we performed proteomic analysis of GBM cells after treatment with TMZ for either one week or two. The remaining cells following TMZ treatment were used for assessment and we were able to identify differentially expressed proteins. Following this, we verified two of these differentially expressed proteins, DHC2 and KIF2B, in cells, nude mouse xenografts and clinical samples. We demonstrated that TMZ induced morphological changes in U87 cells and that these alterations were a result of cytoskeleton re-organization. At the protein level, cytoskeleton-related proteins, such as DHC2 and KIF2B, were deregulated by TMZ treatment. Knockdown of DHC2 expression enhanced sensitivity of U87 and primary GBM cells to TMZ treatment. *Ex vivo* data further confirmed that DHC2 expression in GBM tissue samples was associated with tumor recurrence after TMZ chemotherapy. Thus, our current data demonstrated that cytoskeleton re-organization contributes to TMZ resistance in glioblastoma U87 cells and knockdown of DHC2 expression induced sensitivity of glioblastoma cells to TMZ treatment. Future studies should aim to verify targets of DHC2 expression, in combination with TMZ, as a novel therapeutic strategy to treat GBM.

Our current study assessed the effect of the cytoskeleton inhibitor, cytochalasin D, on sensitivity of U87 cells to TMZ treatment. Cytochalasin D is a mycotoxin and is produced by Helminthosporium and other molds. Functionally, cytochalasin D is cell-permeable and a potent inhibitor of actin polymerization, by binding to F-actin polymers and preventing polymerization of actin monomers^16^. We did not demonstrate a synergistic effect of TMZ with cytochalasin B, another type of cytochalasin. Cytochalasin B can inhibit cytoplasmic division by blocking the formation of contractile microfilaments. Cytochalasin B also inhibits cell mobility and induces nuclear extrusion. Cytochalasin B shortens actin filaments by blocking monomer addition to the growing end of the polymer chain^17^. The differences between the interactions of microfilaments with CCB/CCD may result in different effects when combined with TMZ in GBM cells. Nocodazole, an anti-neoplastic agent, exerts its effect by interfering with polymerization of microtubules in cells. When combined with TMZ, we were unable to demonstrate any synergistic effect. Further studies are needed to elucidate how TMZ affects the reorganization of microfilaments and microtubules in GBM cells.

DHC2 and KIF2B function in the rearrangement and degradation of the cytoskeleton and mitosis in cells. DHC2 is part of a complex of proteins called dynein-2. Dynein-2 is involved in IFT, by which materials are carried within cilia. In contrast, other motor proteins, namely kinesin, move towards the positively charged end of microtubules. These proteins are known as plus-end directed motors^18^. Anterograde IFT, from the base of the cilium to the distal tip, is powered by the positive-end-directed heterotrimeric kinesin-II, whereas retrograde IFT, from the tip back to the bas, depends on the negative-end-directed dynein motor complex^19^. Dynein can be divided into two groups; cytoplasmic and axonemal dyneins, also known as ciliary and flagellar dyneins, respectively. Cytoplasmic dyneins, including DHC2, are necessary for organelle transportation and centrosome assembly and possesses ability to position cellular organelles^20^ and transport cellular products. During cell division, cytoplasmic dynein plays an important role in moving chromosomes and positioning mitotic spindles^21^,^22^. Cilia cannot be synthesized or maintained without IFT; thus, the latter is necessary for the synthesis of cilia and flagella, and DHC2 plays an important role in retrograde IFT^23^. In our current study, we found that DHC2 expression was up-regulated in GBM cells after TMZ treatment, which is associated with TMZ resistance. Although we were unable to determine how DHC2 mediated TMZ resistance in GBM cells, previous studies have shown that mammalian Hedgehog (Hh) pathway is activated only under conditions in ciliated cells^24^. Hedgehog signaling is essential in vertebrate development^25^ and inappropriate activity of this pathway may lead to development of human cancers^26^. DHC2 has been reported to be important in activation of the mouse Hh pathway, and lack of the normal activity of the IFT retrograde motor in DHC2 mutants was unable to activate Hh pathway correctly^27^. Thus, abnormally activated Hh pathway may promote tumor cells to become resistant to TMZ treatment. Our current data showed that knockdown of DHC2 expression inhibited the formation of the protrusions in GBM cells and induced sensitivity of GBM cells to TMZ treatment. Significantly, expression of DHC2 increased in GBM samples from patients with recurrent tumors. Knockdown of DHC2 expression using siRNA sensitized U87 and primary GBM cells to TMZ. Conversely, DHC2 is necessary for the construction, extension, and maintance of cell protrusions and increased DHC2 expression by TMZ promoted protrusion synthesis, extension and maintenance. This may explain why TMZ can induce changes in cell morphology. Our findings indicate that DHC2 may be a candidate target in therapy for gliomas, especially in combination with TMZ.

However, our current data were unable to confirm whether KIF2B played a role in TMZ-induced chemoresistance. KIF2B is a member of the kinesin family and functions in cytoskeleton organization; thus, it plays an important role in cell division. Kinesin functions by moving along microtubule filaments through hydrolysis of ATP^11^,^12^,^13^. The movement of kinesin is necessary for several cellular activities, including mitosis, meiosis, and transportation of cellular cargo^14^. Most kinesins move towards the positive end of the microtubule, which transport cellular products from the cell center towards the periphery^11^. KIF2B not only localizes to centrosomes and midbodies predominately, but also localizes to spindle microtubules and kinetochores transiently^28^. The temporal regulation of kinetochore-microtubule attachments by KIF2B, CLASP1, and Astrin plays a central role in chromosome segregation during cell division^15^. In our current study, we found that TMZ increased KIF2B expression, but knockdown of KIF2B expression had no impact on sensitivity of GBM cells to TMZ treatment. This may be due to over-expression of KIF2B was one of the results after TMZ treatment but not sufficient to mediation of TMZ resistance.

As a standard treatment for newly diagnosed and recurrent GBMs, TMZ can improve the survival of patients, although TMZ-induced tumor resistant phenotypes are common and may arise from a variety of mechanisms^29^,^30^. The most recognized is through the action of O^6^-methylguanine–DNA methyltransferase (MGMT), a DNA repair enzyme. When highly expressed, MGMT removes the alkyl group from the O^6^-guanine, thereby antagonizing the therapeutic effects of TMZ^31^. Other resistance-related mechanisms include activation of different pro-survival pathway genes such as Akt, NFκB, PI3K, p53, and β-catenin^32^,^33^,^34^,^35^ and deficits in mismatch and base excision repair^36^,^37^. Original or acquired TMZ resistance accounts for the majority of disease progression during treatment or after initial treatment success^38^. Thus, better understanding of the molecular mechanisms underlying TMZ resistance and development of effective therapeutic regimens are currently a major challenge^39^. In our study, cytoskeleton re-organization and up-regulation of DHC2 was demonstrated as novel mechanisms for TMZ resistance in GBM cells. However, the mechanisms of TMZ resistance are likely to be interconnected. Thus, further studies are needed to fully elucidate the pathways by which resistance occurs.

Our data demonstrated that re-organization of cytoskeleton is important in mediating TMZ-resistance of GBM cells. DHC2 may be a suitable molecular target in combination with TMZ, in patients with GBM. However, further studies are needed in order to examine TMZ-induced chemotherapy resistance in GBM cells.

## Materials and Methods

### Cell lines and culture

Human glioma cell lines U87 and U251 were purchased from the Chinese Academy of Sciences (Shanghai, China) and maintained in Dulbecco’s Modified Eagle’s Medium (DMEM glucose 4.5 g/L; Gibco, Carlsbad, CA, USA) with 10% fetal bovine serum (FBS; Gibco), 100 U/mL penicillin, and 100 mg/mL streptomycin (Gibco) at 37 °C in a humidified incubator with 5% CO_2_. In drug treatment experiments, cells were seeded and grown overnight and changed to DMEM containing 200 μM TMZ (Sigma-Aldrich, St. Louis, MO, USA) or dimethyl sulfoxide (DMSO; Sigma-Aldrich) for up to two weeks.

Two primary cell lines, Primary-1^#^ (PRI-1) (derived from a 43 year-old female patient), Primary-2^#^ (PRI-2) (derived from a 52 year-old male patient) were established from tumor tissues and cultured *in vitro* culture. Tumor tissues were cut into very small fragments and then digested with 0.25% trypsin (Gibco). After filtration, non-adherent cells were maintained in DMEM (glucose 4.5 g/L; Gibco) with 10% FBS (Gibco) in a humidified incubator at 37 °C with 5% CO_2_. After identification, primary cells were used in this study, passaged or stored under cryopreservation.

### Flow cytometric cell cycle assay

To assess changes in cell cycle distribution following TMZ treatment, cells were seeded in 6-well plates and cultured in DMEM containing 10% FBS and treated with 200 μM TMZ (Sigma-Aldrich) or DMSO for 48 h. At the end of experiments, a total of 5 × 10^6^ cells were harvested, rinsed with iced-cold phosphate-buffered saline (PBS), and fixed with 70% ice-cold ethanol for 48 h at 4 °C. Fixed cells were then rinsed with cold PBS, followed by incubation with PBS containing 10 μg/mL propidium iodide and 0.5 μg/mL RNAase for 15 min at 37 °C. The DNA content of the labeled cells was analyzed using flow cytometric analysis using a FACS Caliber instrument (BD Biosciences, San Jose, CA, USA).

### Two-dimensional gel electrophoresis

GBM U87 cells were treated with 200 μM of TMZ or DMSO for one week or two and subjected to total cellular proteins extraction using lysis buffer containing 4% (w/v) CHAPS (Sigma-Aldrich), 8 M urea (GE Healthcare, Pittsburgh, PA, USA), 1% (v/v) Pharmalytes (GE Healthcare), 2 mg/mL DTT (Sigma-Aldrich), and trace amounts of bromophenol blue (Sigma-Aldrich) followed by sonication on ice. Next, lysates were centrifuged at 12,000 rpm for 1 h at 4 °C. Subsequently, protein concentration of supernatants was determined by the modified Bradford method, and aliquots of protein samples were stored at −80 °C. Differentially expressed proteins were separated using two dimensional gel electrophoresis (2-DE) and mass spectrometry. The 2-DE was performed using 4–7 pI immobiline strips and gels were then subjected to silver staining to visualize proteins. Protien spots of interest were dissected from the gels and the subjected to Matrix-Assisted Laser Desorption Ionization Time-of-Flight Mass Spectrometry (MALDI-TOF/TOF MS). Experiments were repeated three times.

### MALDI-TOF/TOF MS analysis

For MALDI-TOF/TOF MS analysis, differential in-gel digestion of proteins from gels was carried out and the resulting peptide mixtures were solubilized with 0.1% TFA (a saturated a-cyano-4-hydroxycinnamic acid solution in 0.1% TFA/50% acetonitrile) as the matrix and analyzed in a 4800 MALDI-TOF/TOFTM Analyzer (Applied Biosystems, Foster city, CA, USA). Mass spectra were internally calibrated with calibration mixture [des-Arg1-Bradykinin; Angiotensin I; Glu1-Fibrinopeptide B; ACTH (1–17 clip); ACTH (18–39 clip); and ACTH (7–38 clip)]. Protein identification of the peptide mass fingerprinting from these experiments was performed using the MASCOT search engine (http://www.matrixscience.com, MatrixSicence Ltd., London, UK) against the MSDB (Swiss-Prot or NCBI) protein database. The error tolerance of peptide masses was in the range of 50 ppm. One missed tryptic cleavage site per peptide was allowed during the search. Proteins matching more than four peptides and with a MASCOT score higher than 60 were considered to be significant (p < 0.05). Carboamidomethylation of cysteine was selected as the fixed modification and oxidation of methionine as the variable modification. Protein identification results were filtered with GPS software.

### Bioinformatical analysis

Identified proteins, which were up-, or down regulated after TMZ treatment were subjected to the Enrichment analysis (beta) server of Gene ontology analysis (http://geneontology.org/). The software mapped each of the protein to the repository of information in the base. The dataset was analyzed using the Core Analysis module to rank the proteins into top molecular functions (MF) and cellular component (CC).

### RNA isolation and qRT-PCR

To validate the level of gene expression, qPCR was performed. Total cellular RNA from GBM U87 cells was isolated using Trizol (Invitrogen, Carlsbad, CA, USA) and RNA samples (1 μg per sample) were reversely transcribed into cDNA using the PrimeScript (Takara, Dalian, China) according to the manufacturers’ instructions. The resulting cDNA samples were used as templates for qPCR amplifications using the Maxima SYBR Green/ROX qPCR Master Mix (Thermo Scientific, Pittsburgh, PA, USA). The experiments were performed in triplicate and primer sequences in Sup Table 1.

### Immunofluorescence

Cells, at a density of 1 × 10^5^, were seeded on 35-mm dishes with 10-mm glass bottom (Corning, Corning, NY, USA) and cultured overnight. On the following day, cells were treated with 200 μM TMZ for up to two weeks with a medium change every three days. Cells were then washed with PBS twice and fixed in 4% paraformaldehyde and permeabilized in 0.2% Triton X-100 in PBS at the room temperature. Cells were then incubated with a blocking solution (10% normal serum in PBS) for 30 min and then further incubated with the primary rabbit antibody phospho-H2A.X (Cat #9718; Cell Signaling Technology, Danvers, MA, USA), DHC2 (Cat #ab122525; Abcam, Cambridge, MA,USA) or KIF2B protein (#ab98214; Abcam) all at a dilution of 1:200 in combination with a mouse antibody against β-actin (#3700; Cell Signaling Technology) at a dilution of 1:200, α-Tubulin (#3873; Cell Signaling Technology) at a dilution of 1:50 or phalloidin (#12935; Cell Signaling Technology) for 2 h at 37 °C. Duplicated cells were incubated with a primary rabbit antibody against β-actin (#8480; 1:50, Cell Signaling Technology) and a mouse antibody against α-tubulin (#3873; Cell Signaling Technology) both at a dilution of 1:50 for 2 h at 37 °C. Cells were then washed with PBS twice and incubated with FITC Affinipure goat anti-rabbit IgG (H+L) (#E031220-01, Earthox, Millbrae, CA, USA) at a dilution of 1:100 and Alexa Fluor 555 goat anti-mouse IgG (H+L) (#Z-25105; Invitrogen) at a dilution of 1:1000 for 60 min at 37 °C. Cells were washed with PBS again and stained with 0.2 μg/mL DAPI for 10 min. The cells were reviewed and scored under a confocal laser-scanning microscope (Olympus, FLUOVIEW FV10i, Japan) or dishes were stored at 4 °C in the dark for future review. Fluorescence photometric values of each protein in all images were quantified using the software built in the confocal laser-scanning microscope-computer system.

### siRNA transfection

Cells were transfected with chemical synthetized siRNA purchased from GenePharma Co. (Shanghai, China) using Lipofectamine 2000 reagent (Invitrogen) for 8 h according to the manufacturer’s protocol. siRNA sequences were in Sup Table 1. Cells were then subjected to Western blot analysis of protein expression.

### Protein extraction and Western blot

Cells were lysed in RIPA Buffer (Sigma-Aldrich) and protein concentration was determined by using the BCA assay kit (Sigma-Aldrich). Total protein (40 μg) was resolved by sodium dodecyl sulfate polyacrylamide gel electrophoresis (SDS-PAGE) on a 10% or 5% gel and electrotransferred to polyvinylidene fluoride membranes (Millipore, Billerica, MA, USA). The membranes were then incubated in 5% nonfat dry milk in Tris-buffered saline (TBS, pH 7.6) and then overnight at 4 °C with a rabbit monoclonal anti-DHC2 (#ab122525; Abcam) at a dilution of 1:1000, anti-KIF2B (#ab98214; Abcam) at a dilution of 1:2000 or anti-Phospho-H2A.X (#9718; Cell Signaling Technology) at a dilution of 1:1000. Membranes were then incubated at 37 °C for 1 h with an HRP-conjugated anti-rabbit IgG antibody (#7074; Cell Signaling Technology) at a dilution of 1:2000. After three washes with TBS, membranes were briefly incubated with chemiluminescent HRP substrate (#WBKLS0100; Millipore) and were photo-developed in Image Station 2000 MM (Kodak, Rochester, Minnesota, USA). Quantity One 4.6.2 (Bio-Rad Laboratories, Hercules, CA, USA) was used to quantify the density of the target protein in the membranes.

### Alive cell counting

Cells were seeded in 6-well plates and grown overnight and on the next day, cells were treated with TMZ (200 μM), cytochalasin B (CCB, 1 μg/mL), cytochalasin D (CCD, 0.5 μg/mL), nocodazole (1 μg/mL), or DMSO (all from Sigma-Aldrich) in different combinations and concentrations for up to two weeks. At the end of experiments, cells were digested with 0.25% Trypsin-EDTA (#25200-056, Gibco) and stained with Trypan blue (#T8154, Sigma-Aldrich) and the colorless cells of each group were counted with a cell counting chamber.

### Immunohistochemistry

Nude mouse xenografts (see below) and *ex vivo* GBM tissue samples were subjected to immunohistochemical staining of DHC2 and KIF2B proteins using a standard protocol according to a previous study^40^. DHC2 (#ab122525; Abcam) and KIF2B (#ab98214; Abcam) antibodies were used at dilution of 1:200. The stained tissue sections were reviewed and scored separately by two pathologists blinded to the clinical parameters under a microscope. Expression level of these proteins were assessed by the percentage of positive cells and staining intensity, i.e., the percentage of positive cells was scored as follows: 0% (absent), 1–5% (sporadic), 6–25% (local), 26–50% (occasional), 51–75% (majority), and 76–100% (most). The staining intensity of cancer cells was graded as 0 (no staining), 1 (weak staining, light yellow), 2 (moderate staining, yellowish brown), and 3 (strong staining, brown). An intensity score of 2with at least 50% of positive cells was considered as high expression (or overexpression), and <50% of positive cells or <2 in intensity score was regarded as low expression.

### Nude mouse tumor cell xenograft assay

The protocol for the use of the mouse xenograft model was approved by the Experimental Animal Center of Southern Medical University (Guangzhou, China). In brief, GBM U87 cells were subcutaneously injected in the flank region of nude mice (BALB/c-nu, 21–28 days old). Ten days later, the mice were subjected to daily intraperitoneal injection of TMZ (Merck Co., NJ, USA, 20 mg/kg for each mouse) for two weeks and equal amount of DMSO was used as a vehicle control. Mice were euthanized and tumor xenografts were carefully dissected and tissue samples from the core and peripheral regions were separated for immunohistochemical analysis.

### Measurement of cell length, width and square or protrusion

NIH Image J (NIH, Bethesda, MD, USA. http://rsb.info.nih.gov) to measure cell length, width and square. The length of pseudopodia was measured using NeuronJ plug-in for NIH ImageJ software as described previously^41^. Multiple pseudopodium extending from a single multipolar cell was defined as a group to obtain summated pseudopodium lengths per cell.

### GBM tissue samples

In this study, we also used samples from nine GBM patients with recurrent GBM patients who were treated with TMZ before their second surgery, and 12 samples of primary GBM that were not treated with TMZ from Nanfang hospital (Guangzhou, China). Tissue blocks were retrieved from pathology department and subjected to sectioning and immunohistochemical staining of DHC2 and KIF2B proteins. This study was approved by The Ethics Committee of Nanfang Hospital in accordance with the Helsinki Declaration and patients or guardians signed an inform consent form to participate this study.

### Statistical analyses

All experiments in this study was performed in triplicate and repeated at least once. All data was summarized as means ± SD. While the variance was homogeneity, One-way ANOVA was performed for the differences between groups and the LSD test was applied to compare the two measured groups. While the variance was heterogeneity, Welch test was applied to compare differences between groups and Dunnett’s T3 was applied for two means groups. A *P* value ≤ 0.05 was considered as statistically significant (two-tailed).

## Additional Information

**How to cite this article**: Wang, H. *et al*. Expression of dynein, cytoplasmic 2, heavy chain 1 (DHC2) associated with glioblastoma cell resistance to temozolomide. *Sci. Rep.* **6**, 28948; doi: 10.1038/srep28948 (2016).

## Supplementary Material

Supplementary Information
