# Supplementary material for: Expression of dynein, cytoplasmic 2, heavy chain 1 (DHC2) associated with glioblastoma cell resistance to temozolomide
Source: Sci Rep. 2016 Jul 4;6:28948. doi: 10.1038/srep28948 (PMC4931463; doi:10.1038/srep28948)
Supplement: Supplementary Information [file srep28948-s1.pdf]

Expression of dynein, cytoplasmic 2, heavy chain 1 (DHC2) associated with glioblastoma cell resistance to temozolomide

Running title: DHC2 and TMZ resistance in glioblastoma cells

Hai Wang[1,*], Wenfeng Feng[1,*], Yuntao Lu[1,*], Hezhen Li[1], Wei Xiang[1], ZiyangChen[1], MinyiHe[2], Liang Zhao[3],Xuegang Sun[4], Bingxi Lei[1],Songtao Qi[1], Yawei Liu[1,5]

[1]Department of Neurosurgery, Nanfang Glioma Center, Nanfang Hospital, Southern Medical University, Guangzhou 510515, China

[2]Department of Organ Transplantation, Zhujiang Hospital, Southern Medical University, Guangzhou 510515, China

[3]Departmentof Pathology, Nanfang Hospital, Southern Medical University, Guangzhou 510515, China.

[4]Schoolof Traditional Chinese Medicine, Southern Medical University, Guangzhou 510515,China

[5]Nanfang Neurosurgery Research Institution, Nanfang hospital, Southern Medical University, Guangzhou 510515,China

*These authors contributed equally to this work.

Corresponding author: Prof. Songtao Qi or Yawei Liu, Department of Neurosurgery,

Nanfang Glioma Center, Nanfang Hospital, Southern Medical University, Guangzhou Dadaobei, Guangzhou 510515, China; Tel.: 86-020-61641801; Fax: 86-020-61641801; Email: qisongtaonfyy@126.com for SQ or Tel.: 86-020-61641806; Fax: 86-020-61641806; Email: limpduck@163.com for YL.

Supplemental Tables

Table S1. Primers/siRNA sequences

Gene	Sequence	Purpose
GAPDH	5'-ATCATCAGCAATGCCTCCTG-3'<br>5'-ATGGACTGTGGTCATGAGTC-3'	qPCR
DHC2	5'-TGCTGGTGTTTTTCAAGCTG-3'<br>5'-TTCTGCTCCATTCCTGATCC-3'	qPCR
KIF2B	5'-GGGAAGTGGGAAGACGTACA-3'<br>5'-GATTTGCTGATTGCCATCCT-3'	qPCR
DHC2 siRNA	(1) 5'-ACAGGCUCUUCUCUCUGAATT-3'<br>(2) 5'-GGAAUUGAAUACUCUUCAATT-3'	siRNA
KIF2B siRNA	(1) 5'-CGGCCCAAGAUUGUUCUAATT-3'<br>(2) 5'-GUGGAGUACACCAUGAUAUTT-3'	siRNA
Negative control siRNA	5'-UUCUCCGAACGUGUCACGUTT-3'	siRNA

Table S2. Upregulated expression of proteins in U87 cells after TMZ treatment

**Protein**	**Accession number**	**Gene name**	**MW**	**PI**
BRCA1 associated RING domain 1 variant	Q3B891	BRCA1	53350.61	5.62
Spectrin, alpha, erythrocytic 1	D3DVD8	SPTA1	178905.85	4.67
Microtubule-actin cross-linking factor 1, isoforms 1/2/3/5	Q6IPG6	MACF1	609179.65	5.06
Vimentin	P08670	VIM	53676.1	4.77
cDNA FLJ55253, highly similar to Actin, cytoplasmic 1	B4E3A4		40115.87	5.05
40S ribosomal protein SA (Fragment)	C9J9K3	RPSA	29601.04	4.91
cDNA FLJ53902, highly similar to Homo sapiens kinesin family member 2B (KIF2B)	B4DY48	KIF2B	63984.93	9.12
ATP synthase subunit beta (Fragment)	Q0QEN7	ATP5B	48083.04	4.7
Mitochondrial dynamin-like 120 kDa protein	E5KLM2	OPA1	11165	7.87
Putative uncharacterized protein DKFZp781G125	Q68CP7	DKFZp781G125	135753.44	6.55
Cytoplasmic dynein 2 heavy chain 1	Q8NCM8	DYNC2H1	495789.75	6.5
Myosin-14	F2Z2U8	MYH14	232101	5.3
Glutamine-dependent NAD(+) synthetase (Fragment)	H0YCQ6	NADSYN1	29910	6.61
Poly [ADP-ribose] polymerase 14	Q460N5	PARP14	202800.21	6.81
ZNF557 protein (Fragment)	Q4G137	ZNF557	47290.17	9.53
GRIP and coiled-coil domain-containing protein 2	Q8IWJ2	GCC2	196872.12	4.81
PAPOLG protein (Fragment)	Q2TAI9	PAPOLG	58357.31	8.55
Testis-specific gene 10 protein (Fragment)	B2R8F9	TSGA10	202800.21	6.81
Tubulin alpha-1B chain	P68363	TUBA1B	50803.86	4.7
Actin, cytoplasmic 1	P60709	ACTB	42051.86	5.15
N-acetylated-alpha-linked acidic dipeptidase 2	J3KNJ3	NAALAD2	80284.35	8.67

ZNF234	Q86WM3	ZNF234	16555.82	6.92
Multidrug resistance-associated protein 1	I3L4X2	ABCC1	161588.05	6.72
Ropporin-1B	D6RA85	ROPN1B	11799.2	5.89
PRO1975	Q9UHS8	PRO1975	44346.79	9.43
InaD-like protein	B4DE90	INADL	49063.06	5.23
DSP variant protein	Q4LE79	DSP	267367.31	7.37
Brefeldin A-inhibited guanine nucleotide-exchange protein 2	Q9Y6D5	ARFGEF2	204418.08	6.28
cDNA, FLJ93596, highly similar to Homo sapiens sulfide quinone reductase-like	B2R7T6		50198.89	9.45

Table S3. Downregulated expression of proteins in U87 cells after TMZ treatment

**Protein**	**Accession number**	**Gene name**	**MW**	**PI**
Protein FAM71F1	F8WC78	FAM71F1	16442.47	7.71
Elongation factor 1-delta (Fragment)	E9PK01	EEF1D	28917.64	4.64
Eukaryotic translation initiation factor 3 subunit F	B4DMT5	EIF3F	33333.03	5.32
Eukaryotic initiation factor 4A-I (Fragment)	A8K7F6	EIF4A1	46320.67	5.12
Stomatin-like protein 2, mitochondrial	Q9UJZ1	STOML2	38624.24	7.5
cDNA FLJ75549, highly similar to Homo sapiens ribosomal protein, large, P0 (RPLP0), transcript variant 1, mRNA	A8K4Z4	RPLP0	34388.88	5.77
Eukaryotic translation initiation factor 3 subunit I	Q13347	EIF3I	36877.76	5.38
Isoform 3 of Dystonin	Q03001-3	DST	308958.88	6.24
Chloride intracellular channel protein 4	Q9Y696	CLIC4	28981.83	5.26
Proteasome activator complex subunit 3	P61289	PSME3	29601.6	5.76
6-phosphogluconolactonase	O95336	PGLS	27814.66	5.95
Tubulin-specific chaperone A	E5RIW3	TBCA	10131.03	4.29
Myosin light polypeptide 6	J3KND3	MYL6	17218.26	4.29
Isoform 2 of Gamma-tubulin complex component 2	Q9BSJ2-3	TUBGCP2	88661.56	6.75
REG gamma-3 variant	K9J957	PSME3	26964.19	5.74
Heterogeneous nuclear ribonucleoprotein H	G8JLB6	HNRNPH1	51482.28	6.8
Putative uncharacterized protein DKFZp686A15170 (Fragment)	Q68DG4	DKFZp686A15170	21607.09	7.68
Isoform Short of Adenomatous polyposis coli protein	P25054-2	APC	302307.96	7.82

supplement figure legends

Fig. S1. Effects of temozolomide on regulation of viability and morphology in GBM cells. (a) This histogram showed live cell numbers of U87 and U251 cell line after culture for 1 week or 2 weeks in complete culture medium (Untreated), TMZ as a final concentrations of 200 μM diluted by complete culture medium (TMZ) and DMSO diluted by complete culture medium (DMSO) which was used as the vehicle control for TMZ (***P*< 0.01) . (b) The morphology of U87 and U251 cells changed considerably after culture for 1 week or 2 weeks, in the aforementioned conditions. Images marked as "×200" were the same fields to the frames in the images marked as "×100" . The histogram on right showed the measurement results of length, width, square of whole cell; the square of nucleus; the length of protrusion (#*P*> 0.05; **P*< 0.05;and ***P*< 0.01).

Fig. S2. Effects of temozolomide on regulation of cell cycle in GBM cells. The U87 and U251 cells were distributed in various phases throughout the cell cycle after culture in different conditions of temozolomide. The statistical analysis is shown in the right histogram (#*P*> 0.05 and ***P*< 0.01).

Fig. S3. Cytochalasin D (CCD) increased sensitivity of U87 cells to TMZ. (a) After culturing U87 cells with TMZ/DMSO/complete culture medium (Untreated)/CCB/CCD/nocodazole only and/or correlation of two of them for 1 week or 2 weeks. Cell viability and modality change was observed. Imagesmarked as "×200"

were the same fields to the frames in the images marked as “×100”. (b) The lower histogram showed the numbers of survival cells in different groups (#$P > 0.05$; *$P < 0.05$; and **$P < 0.01$).

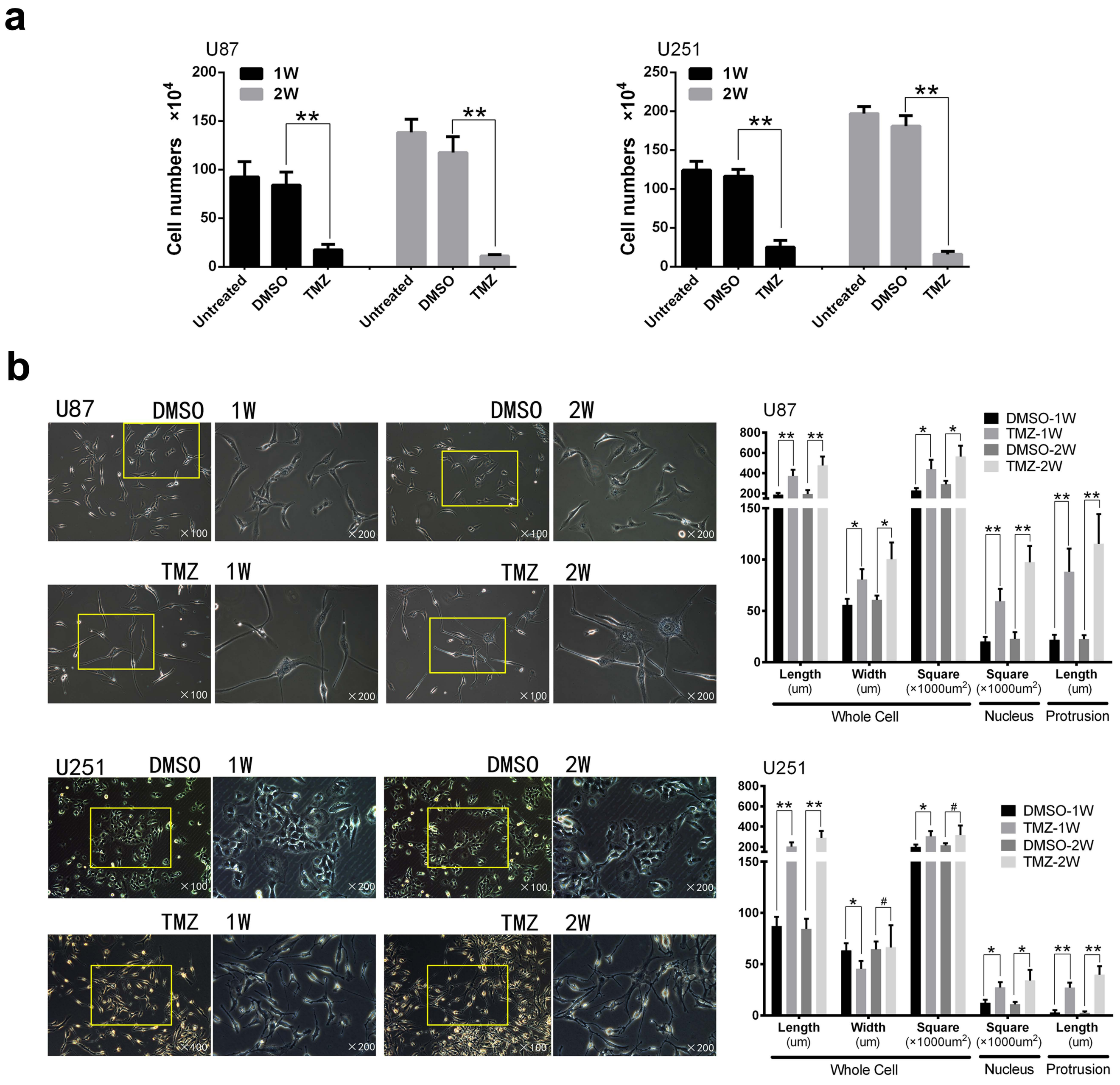
a
U87
U251
1W
2W
Cell numbers ×10^4
**
Untreated
DMSO
TMZ
b
U87
U251
DMSO 1W
DMSO 2W
TMZ 1W
TMZ 2W
×100
×200
DMSO-1W
TMZ-1W
DMSO-2W
TMZ-2W
Length (um)
Width (um)
Square (×1000um2)
Whole Cell
Nucleus
Protrusion
#

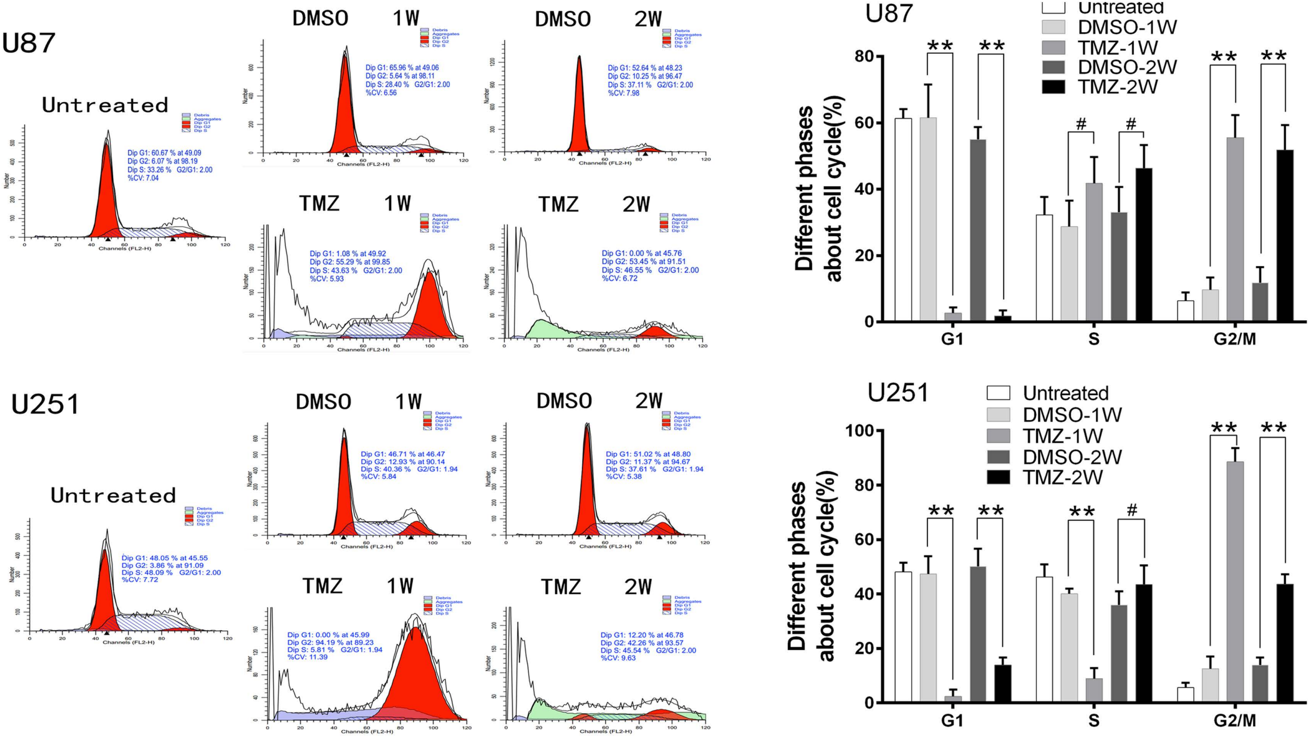

U87
Untreated
Dip G1: 60.67 % at 49.09
Dip G2: 6.07 % at 98.19
Dip S: 33.26 % G2/G1: 2.00
%CV: 7.04
DMSO 1W
Dip G1: 65.96 % at 49.06
Dip G2: 5.64 % at 98.11
Dip S: 28.40 % G2/G1: 2.00
%CV: 6.56
DMSO 2W
Dip G1: 52.64 % at 48.23
Dip G2: 10.25 % at 96.47
Dip S: 37.11 % G2/G1: 2.00
%CV: 7.98
TMZ 1W
Dip G1: 1.08 % at 49.92
Dip G2: 55.29 % at 99.85
Dip S: 43.63 % G2/G1: 2.00
%CV: 5.93
TMZ 2W
Dip G1: 0.00 % at 45.76
Dip G2: 53.45 % at 91.51
Dip S: 46.55 % G2/G1: 2.00
%CV: 6.72
Channels (FL2-H)
Number
Debris
Aggregates
Dip G1
Dip G2
Dip S
U251
Untreated
Dip G1: 48.05 % at 45.55
Dip G2: 3.86 % at 91.09
Dip S: 48.09 % G2/G1: 2.00
%CV: 7.72
DMSO 1W
Dip G1: 46.71 % at 46.47
Dip G2: 12.93 % at 90.14
Dip S: 40.36 % G2/G1: 1.94
%CV: 5.84
DMSO 2W
Dip G1: 51.02 % at 48.80
Dip G2: 11.37 % at 94.67
Dip S: 37.61 % G2/G1: 1.94
%CV: 5.38
TMZ 1W
Dip G1: 0.00 % at 45.99
Dip G2: 94.19 % at 89.23
Dip S: 5.81 % G2/G1: 1.94
%CV: 11.39
TMZ 2W
Dip G1: 12.20 % at 46.78
Dip G2: 42.26 % at 93.57
Dip S: 45.54 % G2/G1: 2.00
%CV: 9.63
U87
Untreated
DMSO-1W
TMZ-1W
DMSO-2W
TMZ-2W
Different phases about cell cycle(%)
G1
S
G2/M
**
#
U251
Untreated
DMSO-1W
TMZ-1W
DMSO-2W
TMZ-2W
Different phases about cell cycle(%)
G1
S
G2/M
**
#

a

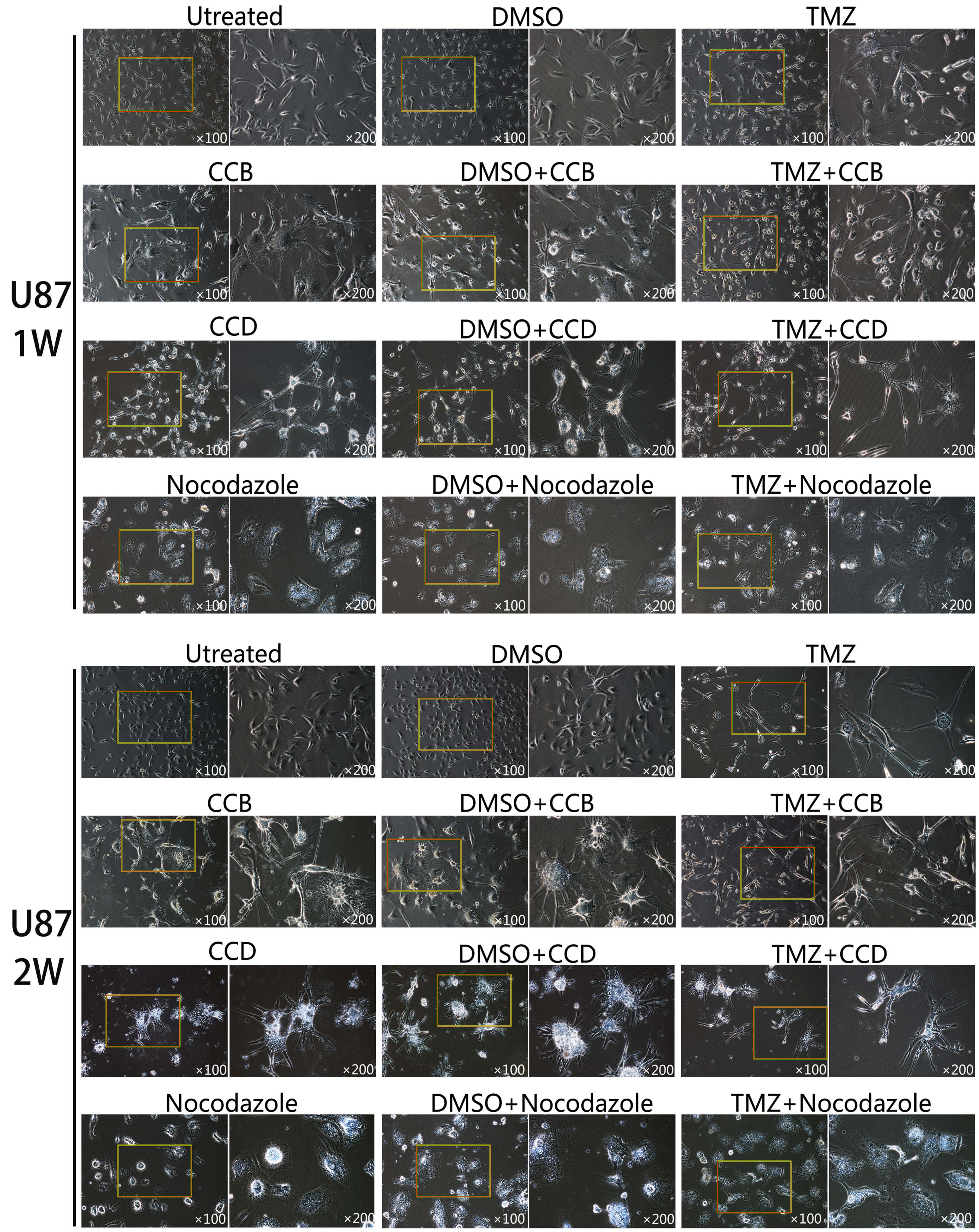
Utreated
DMSO
TMZ
CCB
DMSO+CCB
TMZ+CCB
CCD
DMSO+CCD
TMZ+CCD
Nocodazole
DMSO+Nocodazole
TMZ+Nocodazole
U87 1W
U87 2W
×100
×200

b

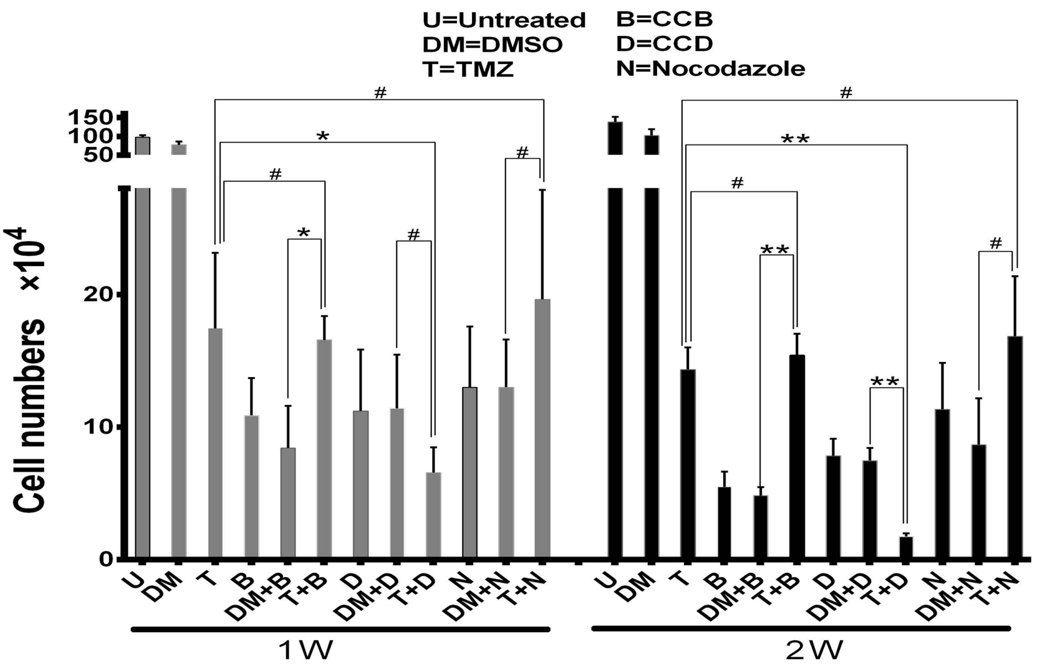
U=Untreated
DM=DMSO
T=TMZ
B=CCB
D=CCD
N=Nocodazole
Cell numbers ×10^4
150
100
50
20
10
0
#
*
**
U
DM
T
B
DM+B
T+B
D
DM+D
T+D
N
DM+N
T+N
1W
2W
